# *N*′-(5-Chloro-2-hy­droxy­benzyl­idene)-4-meth­oxy­benzohydrazide

**DOI:** 10.1107/S1600536812010707

**Published:** 2012-03-17

**Authors:** Yao Tan

**Affiliations:** a School of Chemistry and Environmental Engineering, Chongqing Three Gorges University, Chongqing 404000, People’s Republic of China

## Abstract

The asymmetric unit of the title compound, C_15_H_13_ClN_2_O_3_, contains two independent hydrazone mol­ecules. Each mol­ecule adopts an *E* configuration with respect to the methyl­idene unit and forms an intra­molecular O—H⋯N hydrogen bond. The principal difference between the two unique mol­ecules is the relative orientation of the two benzene rings, the dihedral angles between them being 4.0 (3) and 65.9 (3)°, respectively. In the crystal, mol­ecules are linked through N—H⋯O hydrogen bonds, forming chains running along the *c* axis.

## Related literature

For similar hydrazone derivatives, see: Li (2012 ▶); Zhu *et al.* (2012 ▶); Shen *et al.* (2012 ▶); Liu *et al.* (2011 ▶); Lei (2011 ▶).
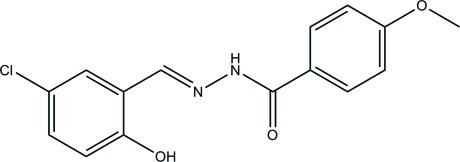


## Experimental

### 

#### Crystal data

C_15_H_13_ClN_2_O_3_
*M* *_r_* = 304.72
Monoclinic, 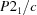

*a* = 17.569 (3) Å
*b* = 8.367 (2) Å
*c* = 19.454 (3) Å
β = 93.683 (3)°
*V* = 2853.8 (9) Å^3^
*Z* = 8
Mo *K*α radiation
μ = 0.28 mm^−1^
*T* = 298 K
0.11 × 0.08 × 0.07 mm

#### Data collection

Bruker SMART CCD area detector diffractometer
Absorption correction: multi-scan (*SADABS*; Bruker, 2000 ▶) *T*_min_ = 0.970, *T*_max_ = 0.981
11127 measured reflections
3752 independent reflections
2545 reflections with *I* > 2σ(*I*)
*R*_int_ = 0.031
θ_max_ = 22.7°

#### Refinement

*R*[*F* ^2^ > 2σ(*F* ^2^)] = 0.038
*wR*(*F* ^2^) = 0.105
*S* = 1.02
3752 reflections
389 parameters
2 restraints
H atoms treated by a mixture of independent and constrained refinement
Δρ_max_ = 0.15 e Å^−3^
Δρ_min_ = −0.23 e Å^−3^

### 

Data collection: *SMART* (Bruker, 2000 ▶); cell refinement: *SAINT* (Bruker, 2000 ▶); data reduction: *SAINT*; program(s) used to solve structure: *SHELXS97* (Sheldrick, 2008 ▶); program(s) used to refine structure: *SHELXL97* (Sheldrick, 2008 ▶); molecular graphics: *SHELXTL* (Sheldrick, 2008 ▶); software used to prepare material for publication: *SHELXTL*.

## Supplementary Material

Crystal structure: contains datablock(s) global, I. DOI: 10.1107/S1600536812010707/sj5210sup1.cif

Structure factors: contains datablock(s) I. DOI: 10.1107/S1600536812010707/sj5210Isup2.hkl

Supplementary material file. DOI: 10.1107/S1600536812010707/sj5210Isup3.cml

Additional supplementary materials:  crystallographic information; 3D view; checkCIF report

## Figures and Tables

**Table 1:** Hydrogen-bond geometry (Å, °)

*D*—H⋯*A*	*D*—H	H⋯*A*	*D*⋯*A*	*D*—H⋯*A*
N4—H4*B*⋯O2^i^	0.89 (1)	1.98 (1)	2.843 (3)	164 (2)
N2—H2⋯O5	0.89 (1)	2.01 (1)	2.883 (3)	166 (2)
O4—H4⋯N3	0.82	1.82	2.540 (3)	145
O1—H1⋯N1	0.82	1.80	2.526 (3)	146

Symmetry code: (i) 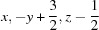.

**Fig. 1.**
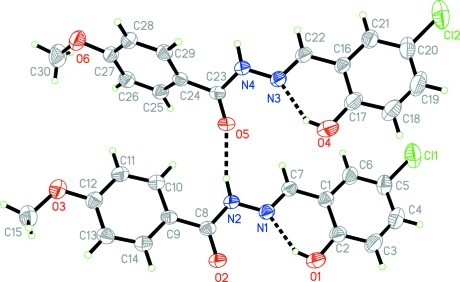
The molecular structure of (I) with 30% probability displacement ellipsoids. Hydrogen bonds are shown as dashed lines.

**Fig. 2.**
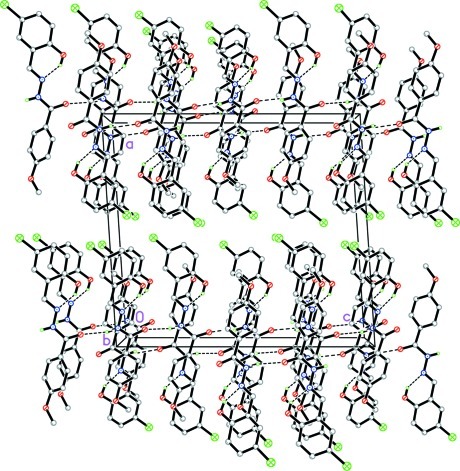
The chain stucture of (I), viewed along the b axis. Hydrogen bonds are shown as dashed lines.

## supplementary crystallographic information

### Comment

As an extension of the work on the structures of hydrazone derivatives (Li, 2012; Zhu *et al.*, 2012; Shen *et al.*, 2012; Liu *et al.*, 2011; Lei, 2011), the author reports here the structure of a new benzohydrazide compound, (I). The asymmetric unit of (I) contains two independent hydrazone molecules, Fig 1. Each molecule adopts a *trans* configuration with respect to the methylidene unit. The dihedral angles between the C1—C6 and C9—C14 benzene rings is 4.0 (3)°. The dihedral angles between the C16—C21 and C24—C29 benzene rings is 65.9 (3)°. In the crystal, molecules are linked through N—H···O hydrogen bonds (Table 1), to form chains running along the *c*-axis (Fig. 2).

### Experimental

4-Methoxybenzohydrazide (0.1 mmol, 16.6 mg) and 5-chloro-2-hydroxybenzaldehyde (0.1 mmol, 15.6 mg) were dissolved in methanol (30 ml). The reaction mixture was heated under reflux for 30 min and cooled gradually to room temperature. Thin, colourless needle-like crystals were obtained by slow evaporation of the solution containing the compound in air.

### Refinement

H2 and H4B were located in a difference Fourier map and refined isotropically, with N—H distances restrained to 0.90 (1) Å. The remaining hydrogen atoms were positioned geometrically and constrained to ride on their parent atoms, with C—H = 0.93–0.96 Å, and with *U*_iso_(H) = 1.2*U*_eq_(C) and 1.5*U*_eq_(O1, O4, C15, and C30). Crystals were very small and weakly diffracting with no useful data observed beyond theta = 22.66°.

### Crystal data

**Table:**

C_15_H_13_ClN_2_O_3_	*F*(000) = 1264
*M**_r_* = 304.72	*D*_x_ = 1.418 Mg m^−^^3^
Monoclinic, *P*2_1_/*c*	Mo *K*α radiation, λ = 0.71073 Å
Hall symbol: -P 2ybc	Cell parameters from 2580 reflections
*a* = 17.569 (3) Å	θ = 2.3–24.1°
*b* = 8.367 (2) Å	µ = 0.28 mm^−^^1^
*c* = 19.454 (3) Å	*T* = 298 K
β = 93.683 (3)°	Cut from a needle, colorless
*V* = 2853.8 (9) Å^3^	0.11 × 0.08 × 0.07 mm
*Z* = 8	

### Data collection

**Table:**

Bruker SMART CCD area detector diffractometer	3752 independent reflections
Radiation source: fine-focus sealed tube	2545 reflections with *I* > 2σ(*I*)
Graphite monochromator	*R*_int_ = 0.031
ω scans	θ_max_ = 22.7°, θ_min_ = 2.1°
Absorption correction: multi-scan (*SADABS*; Bruker, 2000)	*h* = −18→18
*T*_min_ = 0.970, *T*_max_ = 0.981	*k* = −7→9
11127 measured reflections	*l* = −21→20

### Refinement

**Table:**

Refinement on *F*^2^	Primary atom site location: structure-invariant direct methods
Least-squares matrix: full	Secondary atom site location: difference Fourier map
*R*[*F*^2^ > 2σ(*F*^2^)] = 0.038	Hydrogen site location: inferred from neighbouring sites
*wR*(*F*^2^) = 0.105	H atoms treated by a mixture of independent and constrained refinement
*S* = 1.02	*w* = 1/[σ^2^(*F*_o_^2^) + (0.0449*P*)^2^ + 0.5824*P*] where *P* = (*F*_o_^2^ + 2*F*_c_^2^)/3
3752 reflections	(Δ/σ)_max_ = 0.001
389 parameters	Δρ_max_ = 0.15 e Å^−^^3^
2 restraints	Δρ_min_ = −0.23 e Å^−^^3^

### Special details

**Table:**

Geometry. All e.s.d.'s (except the e.s.d. in the dihedral angle between two l.s. planes) are estimated using the full covariance matrix. The cell e.s.d.'s are taken into account individually in the estimation of e.s.d.'s in distances, angles and torsion angles; correlations between e.s.d.'s in cell parameters are only used when they are defined by crystal symmetry. An approximate (isotropic) treatment of cell e.s.d.'s is used for estimating e.s.d.'s involving l.s. planes.
Refinement. Refinement of *F*^2^ against ALL reflections. The weighted *R*-factor *wR* and goodness of fit *S* are based on *F*^2^, conventional *R*-factors *R* are based on *F*, with *F* set to zero for negative *F*^2^. The threshold expression of *F*^2^ > σ(*F*^2^) is used only for calculating *R*-factors(gt) *etc*. and is not relevant to the choice of reflections for refinement. *R*-factors based on *F*^2^ are statistically about twice as large as those based on *F*, and *R*- factors based on ALL data will be even larger.

### Fractional atomic coordinates and isotropic or equivalent isotropic displacement parameters (Å^2^)

**Table:**

	*x*	*y*	*z*	*U*_iso_*/*U*_eq_	
Cl1	0.43413 (5)	0.26184 (11)	0.43593 (6)	0.1157 (4)	
Cl2	0.46637 (5)	0.46291 (14)	0.15497 (6)	0.1363 (4)	
N1	0.14645 (12)	0.6342 (2)	0.52429 (10)	0.0574 (5)	
N2	0.07370 (12)	0.6759 (3)	0.50355 (10)	0.0576 (6)	
N3	0.15526 (12)	0.6563 (2)	0.26936 (11)	0.0604 (6)	
N4	0.07935 (12)	0.6606 (3)	0.24935 (10)	0.0600 (6)	
O1	0.25873 (11)	0.6596 (3)	0.61141 (10)	0.0842 (6)	
H1	0.2159	0.6780	0.5939	0.126*	
O2	0.06353 (10)	0.7996 (2)	0.60402 (9)	0.0713 (5)	
O3	−0.26093 (11)	0.9580 (2)	0.46447 (10)	0.0847 (6)	
O4	0.26641 (13)	0.7120 (3)	0.35609 (11)	0.1122 (8)	
H4	0.2214	0.7009	0.3429	0.168*	
O5	0.04802 (10)	0.6214 (2)	0.35756 (9)	0.0716 (5)	
O6	−0.27527 (11)	0.6996 (3)	0.19814 (10)	0.0878 (6)	
C1	0.26287 (14)	0.5095 (3)	0.50765 (14)	0.0598 (7)	
C2	0.29662 (16)	0.5671 (3)	0.56912 (15)	0.0680 (8)	
C3	0.37158 (18)	0.5317 (4)	0.58736 (17)	0.0892 (10)	
H3	0.3944	0.5729	0.6280	0.107*	
C4	0.41271 (18)	0.4375 (4)	0.5469 (2)	0.0911 (10)	
H4A	0.4631	0.4129	0.5602	0.109*	
C5	0.38012 (16)	0.3790 (4)	0.48679 (18)	0.0792 (9)	
C6	0.30662 (15)	0.4144 (3)	0.46739 (15)	0.0732 (8)	
H6	0.2851	0.3739	0.4261	0.088*	
C7	0.18546 (14)	0.5496 (3)	0.48577 (14)	0.0610 (7)	
H7	0.1643	0.5136	0.4436	0.073*	
C8	0.03506 (14)	0.7664 (3)	0.54710 (14)	0.0549 (6)	
C9	−0.04190 (14)	0.8190 (3)	0.52330 (12)	0.0517 (6)	
C10	−0.07920 (15)	0.7694 (3)	0.46248 (13)	0.0648 (7)	
H10	−0.0546	0.7010	0.4334	0.078*	
C11	−0.15120 (16)	0.8190 (3)	0.44458 (14)	0.0700 (8)	
H11	−0.1753	0.7849	0.4032	0.084*	
C12	−0.18882 (16)	0.9187 (3)	0.48663 (14)	0.0635 (7)	
C13	−0.15250 (16)	0.9716 (3)	0.54685 (13)	0.0663 (7)	
H13	−0.1770	1.0408	0.5756	0.080*	
C14	−0.08003 (15)	0.9212 (3)	0.56394 (13)	0.0637 (7)	
H14	−0.0555	0.9576	0.6048	0.076*	
C15	−0.30577 (17)	1.0475 (4)	0.50860 (17)	0.0905 (10)	
H15A	−0.3096	0.9908	0.5511	0.136*	
H15B	−0.3559	1.0628	0.4869	0.136*	
H15C	−0.2823	1.1496	0.5177	0.136*	
C16	0.28167 (14)	0.6057 (3)	0.24456 (14)	0.0608 (7)	
C17	0.31081 (17)	0.6535 (4)	0.30840 (17)	0.0820 (9)	
C18	0.3881 (2)	0.6417 (5)	0.3241 (2)	0.1183 (14)	
H18	0.4081	0.6743	0.3672	0.142*	
C19	0.4352 (2)	0.5837 (5)	0.2780 (2)	0.1143 (13)	
H19	0.4873	0.5763	0.2896	0.137*	
C20	0.40683 (16)	0.5361 (4)	0.21483 (19)	0.0863 (10)	
C21	0.33051 (15)	0.5475 (3)	0.19787 (15)	0.0730 (8)	
H21	0.3112	0.5156	0.1544	0.088*	
C22	0.20133 (15)	0.6144 (3)	0.22551 (14)	0.0623 (7)	
H22	0.1833	0.5891	0.1809	0.075*	
C23	0.02814 (14)	0.6472 (3)	0.29745 (14)	0.0549 (6)	
C24	−0.05182 (14)	0.6598 (3)	0.27157 (12)	0.0520 (6)	
C25	−0.10656 (15)	0.5695 (3)	0.30053 (13)	0.0618 (7)	
H25	−0.0924	0.5008	0.3367	0.074*	
C26	−0.18192 (15)	0.5784 (3)	0.27714 (14)	0.0661 (7)	
H26	−0.2182	0.5148	0.2968	0.079*	
C27	−0.20315 (15)	0.6807 (3)	0.22506 (14)	0.0617 (7)	
C28	−0.14915 (16)	0.7721 (3)	0.19611 (14)	0.0664 (7)	
H28	−0.1637	0.8429	0.1608	0.080*	
C29	−0.07463 (14)	0.7606 (3)	0.21823 (13)	0.0584 (7)	
H29	−0.0385	0.8215	0.1972	0.070*	
C30	−0.33470 (17)	0.6190 (5)	0.23012 (19)	0.1119 (12)	
H30A	−0.3272	0.5057	0.2270	0.168*	
H30B	−0.3829	0.6472	0.2073	0.168*	
H30C	−0.3342	0.6498	0.2777	0.168*	
H2	0.0577 (14)	0.658 (3)	0.4600 (7)	0.080*	
H4B	0.0664 (14)	0.662 (3)	0.2044 (6)	0.080*	

### Atomic displacement parameters (Å^2^)

**Table:**

	*U*^11^	*U*^22^	*U*^33^	*U*^12^	*U*^13^	*U*^23^
Cl1	0.0708 (6)	0.1124 (7)	0.1654 (10)	0.0061 (5)	0.0199 (5)	−0.0250 (6)
Cl2	0.0713 (6)	0.1579 (10)	0.1830 (11)	0.0187 (6)	0.0332 (6)	0.0071 (8)
N1	0.0566 (14)	0.0641 (14)	0.0509 (13)	−0.0031 (11)	0.0003 (11)	0.0036 (11)
N2	0.0580 (14)	0.0720 (15)	0.0426 (13)	−0.0007 (11)	0.0009 (11)	−0.0018 (12)
N3	0.0559 (14)	0.0726 (15)	0.0522 (14)	−0.0024 (11)	−0.0008 (11)	0.0058 (12)
N4	0.0553 (14)	0.0812 (16)	0.0432 (13)	0.0033 (11)	0.0002 (11)	0.0041 (12)
O1	0.0845 (14)	0.1094 (17)	0.0571 (12)	−0.0066 (13)	−0.0066 (10)	−0.0078 (12)
O2	0.0685 (12)	0.0989 (14)	0.0459 (11)	−0.0023 (10)	−0.0005 (9)	−0.0081 (10)
O3	0.0774 (14)	0.0921 (15)	0.0832 (14)	0.0190 (11)	−0.0061 (11)	−0.0025 (12)
O4	0.0952 (17)	0.176 (2)	0.0638 (14)	−0.0141 (17)	−0.0116 (13)	−0.0161 (15)
O5	0.0738 (12)	0.1003 (15)	0.0402 (11)	0.0095 (10)	0.0010 (9)	−0.0005 (10)
O6	0.0610 (13)	0.1084 (16)	0.0922 (15)	0.0039 (11)	−0.0086 (11)	0.0064 (12)
C1	0.0575 (17)	0.0615 (17)	0.0598 (18)	−0.0100 (14)	0.0004 (14)	0.0032 (14)
C2	0.069 (2)	0.076 (2)	0.0587 (19)	−0.0064 (15)	0.0001 (16)	0.0029 (16)
C3	0.073 (2)	0.113 (3)	0.078 (2)	−0.011 (2)	−0.0167 (18)	0.006 (2)
C4	0.062 (2)	0.102 (3)	0.107 (3)	−0.0021 (19)	−0.010 (2)	0.015 (2)
C5	0.0587 (19)	0.077 (2)	0.102 (3)	−0.0065 (15)	0.0061 (17)	−0.0010 (19)
C6	0.0585 (18)	0.077 (2)	0.083 (2)	−0.0081 (15)	0.0012 (16)	−0.0086 (17)
C7	0.0579 (17)	0.0694 (18)	0.0554 (17)	−0.0076 (14)	0.0006 (14)	−0.0057 (15)
C8	0.0615 (17)	0.0606 (17)	0.0430 (16)	−0.0097 (13)	0.0067 (14)	0.0054 (14)
C9	0.0626 (17)	0.0521 (15)	0.0409 (15)	−0.0071 (13)	0.0079 (13)	0.0046 (12)
C10	0.0662 (18)	0.0723 (19)	0.0554 (18)	0.0018 (14)	0.0006 (14)	−0.0087 (14)
C11	0.075 (2)	0.0770 (19)	0.0571 (18)	0.0035 (16)	−0.0060 (15)	−0.0107 (15)
C12	0.0667 (19)	0.0624 (18)	0.0609 (19)	0.0016 (14)	0.0005 (15)	0.0101 (15)
C13	0.081 (2)	0.0647 (18)	0.0543 (18)	0.0082 (15)	0.0094 (15)	0.0012 (14)
C14	0.074 (2)	0.0667 (18)	0.0499 (17)	−0.0009 (15)	0.0025 (14)	0.0029 (14)
C15	0.081 (2)	0.089 (2)	0.102 (3)	0.0167 (18)	0.0116 (19)	0.006 (2)
C16	0.0530 (17)	0.0727 (18)	0.0556 (18)	−0.0054 (13)	−0.0043 (14)	0.0155 (15)
C17	0.067 (2)	0.110 (3)	0.067 (2)	−0.0139 (18)	−0.0104 (17)	0.0100 (19)
C18	0.076 (3)	0.182 (4)	0.093 (3)	−0.019 (3)	−0.026 (2)	0.008 (3)
C19	0.058 (2)	0.153 (4)	0.128 (4)	−0.011 (2)	−0.021 (2)	0.025 (3)
C20	0.0536 (19)	0.097 (2)	0.108 (3)	0.0026 (16)	0.0074 (19)	0.026 (2)
C21	0.0596 (19)	0.084 (2)	0.075 (2)	−0.0016 (15)	−0.0003 (16)	0.0158 (17)
C22	0.0597 (17)	0.0742 (19)	0.0520 (17)	−0.0034 (14)	−0.0034 (14)	0.0081 (14)
C23	0.0652 (18)	0.0570 (16)	0.0426 (16)	0.0010 (13)	0.0037 (14)	−0.0039 (13)
C24	0.0594 (16)	0.0518 (16)	0.0451 (15)	0.0052 (13)	0.0059 (12)	−0.0032 (13)
C25	0.0703 (19)	0.0636 (18)	0.0516 (17)	0.0030 (14)	0.0035 (14)	0.0052 (14)
C26	0.0650 (19)	0.0663 (19)	0.0678 (19)	−0.0045 (14)	0.0088 (15)	−0.0001 (16)
C27	0.0582 (18)	0.0659 (18)	0.0603 (18)	0.0050 (15)	−0.0009 (14)	−0.0055 (15)
C28	0.0682 (19)	0.0692 (19)	0.0612 (18)	0.0134 (15)	−0.0009 (15)	0.0100 (15)
C29	0.0637 (18)	0.0559 (16)	0.0561 (17)	0.0043 (13)	0.0089 (13)	0.0049 (14)
C30	0.060 (2)	0.139 (3)	0.137 (3)	−0.012 (2)	0.006 (2)	0.009 (3)

### Geometric parameters (Å, º)

**Table:**

Cl1—C5	1.721 (3)	C10—H10	0.9300
Cl2—C20	1.727 (3)	C11—C12	1.368 (4)
N1—C7	1.264 (3)	C11—H11	0.9300
N1—N2	1.361 (3)	C12—C13	1.371 (4)
N2—C8	1.351 (3)	C13—C14	1.362 (3)
N2—H2	0.888 (10)	C13—H13	0.9300
N3—C22	1.263 (3)	C14—H14	0.9300
N3—N4	1.366 (3)	C15—H15A	0.9600
N4—C23	1.344 (3)	C15—H15B	0.9600
N4—H4B	0.889 (10)	C15—H15C	0.9600
O1—C2	1.338 (3)	C16—C17	1.372 (4)
O1—H1	0.8200	C16—C21	1.378 (4)
O2—C8	1.217 (3)	C16—C22	1.438 (3)
O3—C12	1.352 (3)	C17—C18	1.375 (4)
O3—C15	1.416 (3)	C18—C19	1.350 (5)
O4—C17	1.343 (4)	C18—H18	0.9300
O4—H4	0.8200	C19—C20	1.356 (5)
O5—C23	1.218 (3)	C19—H19	0.9300
O6—C27	1.349 (3)	C20—C21	1.364 (4)
O6—C30	1.420 (3)	C21—H21	0.9300
C1—C6	1.383 (4)	C22—H22	0.9300
C1—C2	1.387 (4)	C23—C24	1.465 (3)
C1—C7	1.438 (3)	C24—C25	1.372 (3)
C2—C3	1.374 (4)	C24—C29	1.377 (3)
C3—C4	1.355 (4)	C25—C26	1.374 (3)
C3—H3	0.9300	C25—H25	0.9300
C4—C5	1.359 (4)	C26—C27	1.360 (4)
C4—H4A	0.9300	C26—H26	0.9300
C5—C6	1.355 (4)	C27—C28	1.368 (4)
C6—H6	0.9300	C28—C29	1.355 (3)
C7—H7	0.9300	C28—H28	0.9300
C8—C9	1.469 (3)	C29—H29	0.9300
C9—C14	1.368 (3)	C30—H30A	0.9600
C9—C10	1.379 (3)	C30—H30B	0.9600
C10—C11	1.356 (3)	C30—H30C	0.9600
			
C7—N1—N2	120.2 (2)	O3—C15—H15A	109.5
C8—N2—N1	117.2 (2)	O3—C15—H15B	109.5
C8—N2—H2	123.6 (17)	H15A—C15—H15B	109.5
N1—N2—H2	118.2 (17)	O3—C15—H15C	109.5
C22—N3—N4	118.1 (2)	H15A—C15—H15C	109.5
C23—N4—N3	119.0 (2)	H15B—C15—H15C	109.5
C23—N4—H4B	123.0 (17)	C17—C16—C21	119.2 (3)
N3—N4—H4B	117.7 (17)	C17—C16—C22	121.4 (3)
C2—O1—H1	109.5	C21—C16—C22	119.4 (3)
C12—O3—C15	118.8 (2)	O4—C17—C16	122.2 (3)
C17—O4—H4	109.5	O4—C17—C18	118.8 (3)
C27—O6—C30	118.2 (2)	C16—C17—C18	119.0 (3)
C6—C1—C2	117.8 (3)	C19—C18—C17	121.2 (4)
C6—C1—C7	121.1 (3)	C19—C18—H18	119.4
C2—C1—C7	121.2 (3)	C17—C18—H18	119.4
O1—C2—C3	118.2 (3)	C18—C19—C20	120.1 (3)
O1—C2—C1	122.0 (3)	C18—C19—H19	119.9
C3—C2—C1	119.8 (3)	C20—C19—H19	119.9
C4—C3—C2	120.9 (3)	C19—C20—C21	119.9 (3)
C4—C3—H3	119.6	C19—C20—Cl2	120.8 (3)
C2—C3—H3	119.6	C21—C20—Cl2	119.4 (3)
C3—C4—C5	119.9 (3)	C20—C21—C16	120.6 (3)
C3—C4—H4A	120.1	C20—C21—H21	119.7
C5—C4—H4A	120.1	C16—C21—H21	119.7
C6—C5—C4	120.1 (3)	N3—C22—C16	120.1 (2)
C6—C5—Cl1	121.0 (3)	N3—C22—H22	120.0
C4—C5—Cl1	118.8 (3)	C16—C22—H22	120.0
C5—C6—C1	121.5 (3)	O5—C23—N4	121.3 (2)
C5—C6—H6	119.3	O5—C23—C24	123.5 (2)
C1—C6—H6	119.3	N4—C23—C24	115.1 (2)
N1—C7—C1	119.6 (2)	C25—C24—C29	118.0 (2)
N1—C7—H7	120.2	C25—C24—C23	120.1 (2)
C1—C7—H7	120.2	C29—C24—C23	121.9 (2)
O2—C8—N2	120.3 (2)	C24—C25—C26	121.3 (2)
O2—C8—C9	122.3 (2)	C24—C25—H25	119.3
N2—C8—C9	117.4 (2)	C26—C25—H25	119.3
C14—C9—C10	117.4 (2)	C27—C26—C25	119.5 (3)
C14—C9—C8	118.7 (2)	C27—C26—H26	120.2
C10—C9—C8	123.9 (2)	C25—C26—H26	120.2
C11—C10—C9	120.8 (3)	O6—C27—C26	124.7 (3)
C11—C10—H10	119.6	O6—C27—C28	115.7 (3)
C9—C10—H10	119.6	C26—C27—C28	119.6 (2)
C10—C11—C12	120.8 (3)	C29—C28—C27	120.8 (3)
C10—C11—H11	119.6	C29—C28—H28	119.6
C12—C11—H11	119.6	C27—C28—H28	119.6
O3—C12—C11	115.9 (3)	C28—C29—C24	120.8 (2)
O3—C12—C13	124.7 (3)	C28—C29—H29	119.6
C11—C12—C13	119.4 (3)	C24—C29—H29	119.6
C14—C13—C12	119.0 (3)	O6—C30—H30A	109.5
C14—C13—H13	120.5	O6—C30—H30B	109.5
C12—C13—H13	120.5	H30A—C30—H30B	109.5
C13—C14—C9	122.5 (3)	O6—C30—H30C	109.5
C13—C14—H14	118.8	H30A—C30—H30C	109.5
C9—C14—H14	118.8	H30B—C30—H30C	109.5

### Hydrogen-bond geometry (Å, º)

**Table:**

*D*—H···*A*	*D*—H	H···*A*	*D*···*A*	*D*—H···*A*
N4—H4*B*···O2^i^	0.89 (1)	1.98 (1)	2.843 (3)	164 (2)
N2—H2···O5	0.89 (1)	2.01 (1)	2.883 (3)	166 (2)
O4—H4···N3	0.82	1.82	2.540 (3)	145
O1—H1···N1	0.82	1.80	2.526 (3)	146

Symmetry code: (i) *x*, −*y*+3/2, *z*−1/2.
